# Social determinants of antenatal care utilization: an analysis of 2022 Ghana demographic and health survey

**DOI:** 10.1186/s12884-025-08632-7

**Published:** 2026-01-12

**Authors:** Ya Yambao Yang, Joseph Adu

**Affiliations:** 1 Department of Public Health Sciences, University of California, Davis, Davis, CA 95817 USA; 2 Daphe Cockwell School of Nursing, Toronto Metropolitan University, Toronto, ON M5B 2K3 Canada

**Keywords:** Antenatal care, Maternal health, Predisposing factors, Enabling factors, Need factors

## Abstract

**Background:**

Despite Ghana’s free maternal health services policy, antenatal care (ANC) utilization remains suboptimal. This study examines the predisposing, enabling, and need factors associated with ANC use among women in Ghana.

**Methods:**

Using the 2022 Ghana Demographic and Health Survey (GDHS), this study examined 5,302 women aged 15–49 to identify key determinants of ANC use. Antenatal care utilization was defined as completing at least four visits. Guided by the Andersen’s healthcare utilization model, predictors were categorized into predisposing (age, education, marital status, religion), enabling (household income, residence, health insurance), and need factors (self-rated health, pregnancy loss). Descriptive statistics and chi-square tests were used to assess differences in ANC utilization. A modified Poisson regression was applied to estimate adjusted prevalence ratio (aPR) for the association between predisposing, enabling, and need factors and ANC utilization.

**Results:**

Overall, 88.1% of women utilized ANC. Women aged 25–34 were more likely to use ANC compared to those aged 15–24 ( aPR = 1.04; 95% CI: 1.00-1.07). Women with secondary ( aPR = 1.09; 95% CI: 1.05–1.14) and higher education ( aPR = 1.10; 95% CI: 1.04–1.15) were associated with greater ANC use. Married women ( aPR = 1.07; 95% CI: 1.03–1.10) and Muslim women ( aPR = 1.04; 95% CI: 1.01–1.07) were more likely to use ANC, whereas women practicing traditional/other religions were less likely to use ANC ( aPR = 0.83; 95% CI: 0.75–0.92). Women from rich households ( aPR = 1.08; 95% CI: 1.04–1.12) and those with health insurance ( aPR = 1.29; 95% CI: 1.14–1.46) were more likely to use ANC. Residence, self-rated health, and pregnancy loss were not significant predictors.

**Conclusions:**

Key determinants of ANC utilization included age, education, marital status, religion, household income, and health insurance coverage. This suggests that reducing socioeconomic and cultural obstacles is essential for improving maternal health coverage. Future research is needed to understand the indirect barriers that continue to discourage women from seeking ANC in Ghana.

**Supplementary Information:**

The online version contains supplementary material available at 10.1186/s12884-025-08632-7.

## Introduction

Ghana is a West African country with a population of approximately 35 million, of whom 12.2 million are women of reproductive age [1, 2]. In 2008, Ghana implemented a free maternal health services policy under the National Health Insurance Scheme (NHIS) to improve access to care, yet maternal health indicators continue to reflect serious challenges [3]. In 2020, maternal mortality remained high at 263 deaths per 100,000 live births due to pregnancy-related causes in Ghana [4]. One of the significant causes of the high mortality rate is inadequate adherence to the recommended number of antenatal care (ANC) visits [5]. While ANC coverage is high at 88% in Ghana, improved utilization is still needed to reduce maternal and neonatal deaths [6, 7]. ANC refers to the care delivered by skilled health professionals to pregnant women and adolescent girls to promote optimal health outcomes for both mother and child during pregnancy [7, 8]. Antenatal care encompasses a range of services, including vital sign monitoring, urinalysis, and laboratory investigations. Providers also assess fetal well-being by listening to the baby’s heartbeat, offering breastfeeding counselling, and evaluating complications such as vaginal bleeding [7]. Regular ANC enables the early detection of health complications that contribute to maternal morbidity and mortality through regular physical examinations. It further enhances neonatal health by reducing the likelihood of stillbirth, preterm delivery, and low birth weight. To maximize these benefits, at least eight high-quality ANC visits are recommended before delivery to ensure optimal health for the mother and child [7]. Evidence shows that both the frequency and quality of ANC visits are associated with improved maternal health outcomes [6, 7]. Understanding characteristics associated with ANC utilization in Ghana helps reveal the barriers and determinants that contribute to the nation’s elevated maternal mortality rate.

Maternal deaths are not confined to childbirth but may occur before, during, or after delivery. However, most studies have focused on deaths occurring at the point of birth, with less attention given to those arising earlier in pregnancy [9–11]. This research gap underscores the need to examine ANC utilization and the factors associated with it. Previous studies have identified factors such as wealth, age, education, and other socio-demographic characteristics as predictors of ANC utilization [12–14]. However, few studies have explored need factors, such as a woman’s perceived or evaluated health status and pregnancy complications, which influence whether a woman feels medical services are clinically necessary [2, 12, 15]. Expanding the scope of analysis to include these dimensions will provide stronger evidence for designing effective interventions to improve ANC access and reduce maternal deaths.

To fill a critical gap in maternal health research, this study applies the Andersen Behavioral Model of Health Services Utilization to explore the social determinants of ANC utilization among Ghanaian women of reproductive age. The findings may inform strategies to help reduce maternal mortality and ensure that more women receive essential care during pregnancy, thereby safeguarding the health of both mothers and infants.

## Methods

### Study data

With permission from the Monitoring and Evaluation to Assess and Use Results Demographic Health Survey, we analyzed data from the 2022 Ghana Demographic and Health Survey (GDHS) [2]. The survey was carried out by the Ghana Health Service, Ministry of Health, Ghana Statistical Service, and ICF International, and was designed to provide both national and regional estimates. The data collection took place from October 2022 to January 2023. The GDHS provides reliable estimates of health outcomes at the national, regional, and urban-rural levels. A two-stage stratified sampling strategy was used to recruit participants across Ghana’s 16 regions. The GDHS collected information on socio-demographic characteristics and reproductive health topics, including fertility, family planning, and maternal and child health. Data collection included standardized, face-to-face interviewer-administered questionnaires administered in households [2]. The Demographic Health Survey program does not impute missing data. Any missing or “don’t know” responses remain coded as missing in the dataset. After all, no missing data remained in the analytic dataset. In total, 15,014 women aged 15–49 years were interviewed, achieving a response rate of 98%. Among the 15,014 women interviewed, only those who had a live birth in the five years preceding the survey were eligible to answer ANC-related questions. Of these, 5,302 women had completed information on ANC use and exposure variables were included in the analytical sample. This represented 35.3% of the original sample.

### Conceptual framework

**Fig. 1:**
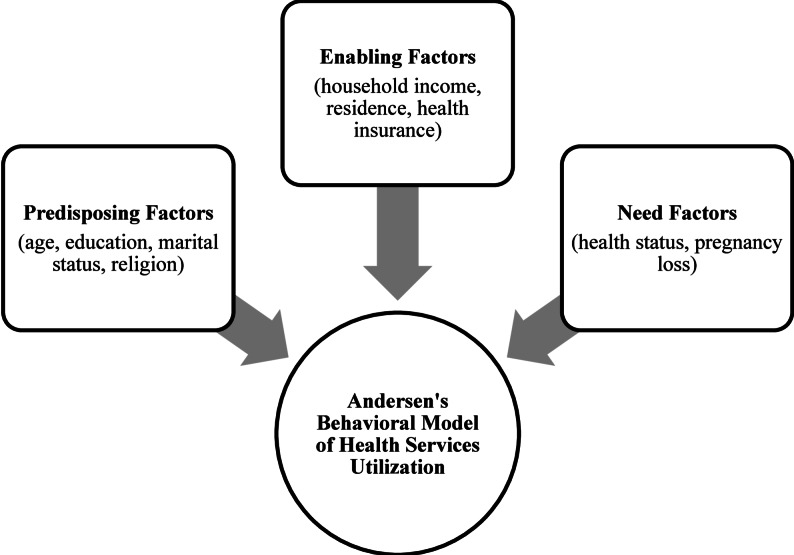
Andersen’s behavioral model of health services utilization

This study adopted and adapted Andersen’s Behavioural Model of Health Services Utilization to analyse ANC use in Ghana [16]. Originally developed in 1968, Andersen’s framework was designed to explain factors influencing the consumption of acute healthcare services. The model identifies determinants of health service utilization at the societal, health system, and individual levels. In our study, emphasis is placed on individual-level factors that shape women’s perceived needs, attitudes, and capacity to seek and access ANC services. These factors are grouped into three categories: predisposing, enabling, and need factors [16, 17]. In Andersen’s framework, predisposing factors capture the demographic and social characteristics that incline individuals toward or away from healthcare use, including age, education, marital status, and religion. Enabling factors refer to the practical resources and community conditions that determine whether care can be accessed. Enabling factors in this study included household income, place of residence, and health insurance. Need factors refer to perceived health conditions that encourage an individual to seek healthcare services. A woman’s perception of her own health can motivate her to seek ANC, and pregnancy loss may increase this perceived vulnerability. Predictors were thus classified as predisposing (age, education, marital status, religion), enabling (household income, residence, and health insurance), and need (health status, pregnancy loss). Please see Fig. 1.

### Outcome variable

Women were considered ANC users if they had completed at least four ANC visits during pregnancy in any setting, whether at home, in public facilities (such as government hospitals, health centers, or mobile clinics), or private facilities (such as clinics, maternity homes, or Planned Parenthood Association of Ghana). Fewer than four visits were coded as non-utilization. The outcome variable was coded as 0 (no ANC use) and 1 (ANC use).

We chose four ANC visits as the minimum threshold because the 2022 Ghana Demographic and Health Survey measures ANC using a visit-based indicator and does not capture the contact-based measures required to operationalize the World Health Organization's 2016 eight-contact recommendation. Visit-based measures quantify the frequency of ANC attendance, whereas contact-based measures reflect whether recommended clinical and counseling components of care were delivered during each encounter.

### Exposure variables

The independent variables included age, education, marital status, religion, household income, residence, health insurance, perceived health status, and pregnancy loss (See Table 1). Maternal age was grouped into three categories: 15–24, 25–34, and 35–49 years. Education was classified as none, primary, secondary, or higher. Marital status was defined as not married or married. Women who have never been in union, lives with a partner but not married, widowed, divorced, and separated are grouped as not married. Religion included Christianity, Islam, and Traditional/Other. Household wealth index was defined as poor, middle, and rich. The household wealth index is a composite measure derived from household income, family assets, and access to water and sanitation. Residence was dichotomized into urban and rural. Perceived health status was measured through self-assessment and categorized as good, moderate, and poor. Pregnancy loss was organized into no loss, one loss, and two or more losses.

**Table 1:** Study variable description

Variable name	Description	Level of measurement
Antenatal care use	Use of antenatal care	0 = Did not use antenatal care
		1 = Used antenatal care
Age	Maternal age (years)	1 = 15 to 24
		2 = 25 to 34
		3 = 35 to 49
Education	Highest educational level	1 = None
		2 = Primary
		3 = Secondary
		4 = High
Marital Status	Current relationship status	0 = Not Married
		2 = Married
Religion	Religious affiliation	1 = Christianity
		2 = Islam
		3 = Traditional/Other
Household Income	Financial status	1 = Poor
		2 = Middle
		3 = Rich
Residence	Place of residence	1 = Urban
		2 = Rural
Health Insurance	Health insurance coverage	0 = No
		1 = Yes
Health Status	Current health status	1 = Good
		2 = Moderate
		3 = Poor
Pregnancy Loss	Number of pregnancy loss	0 = No loss
		1 = 1 loss
		2 = 2 + losses

### Statistical analysis

The GDHS sampling weights were applied to account for the survey’s complex design and to produce population-level estimates. All analyses were conducted in Stata/BE version 18 and incorporated the survey design, including stratification, clustering, and weighting. Descriptive statistics were calculated to summarize the study population, with frequencies and weighted percentages reported. Pearson’s chi-square (χ²) tests were used to assess bivariate relationships between each independent variable and ANC utilization. We employed a modified Poisson regression model with robust error variance to estimate prevalence ratios (PRs) and 95% confidence intervals (CIs) [18]. Although logistic regression is often used for binary outcomes, it yields odds ratios , which can overestimate relative risk when the outcome is common. In contrast, the modified Poisson approach estimates PRs and is therefore more appropriate in this context, given that our outcome was not rare. Both unadjusted and adjusted modified Poisson regression models were fitted to assess the association between predictor variables and ANC utilization. Results are presented as adjusted prevalence ratios (aPRs) for multivariable analyses, each with corresponding 95% CIs. Multicollinearity among predictors was assessed using variance inflation factors (VIFs), and no issues were detected (all VIFs < 2). Statistical significance was defined as *p* < 0.05 (two-sided). Sensitivity analyses were conducted to evaluate the robustness of the findings.

## Results

### Descriptive results

A total of 5,302 women aged 15–49 years responded to the 2022 GDHS question on ANC use. The majority (88.11%) received ANC, while 11.89% did not (See Table 2). Across age groups, ANC use was highest among women aged 25–34 years (48.69%), followed by those aged 35–49 years (26.22%) and 15–24 years (25.09%). By educational status, most women who completed secondary education (54.75%) had used ANC, followed by women with no education (20.06%), primary education (14.88%), and higher education (10.31%). Regarding marital status, the majority of married women (62.46%) had utilized ANC, while only 37.54% of unmarried women had used the service. Regarding religion, most ANC users were Christian (70.52%), followed by Muslim women (25.31%) and those affiliated with Traditional/Other religions (4.16%).

**Table 2:** Distribution of antenatal care use by social determinants (*N* = 5,302)

Predictors	Sample Size for Specific Group	Did Not Use Antenatal Care	Used Antenatal Care	*P*-Value
Total Sample Size				
		668 [11.89%]	4,634 [88.11%]	
Age	5,302 [100.0%]			
15 to 24	1,414 [26.37%]	234 [35.89%]	1,180 [25.09%]	< 0.0001
25 to 34	2,520 [47.49%]	256 [38.62%]	2,264 [48.69%]	
35 to 49	1,368 [26.13%]	178 [25.50%]	1,190 [26.22%]	
Education				
None	1,557 [21.73%]	292 [34.11%]	1,265 [20.06%]	< 0.0001
Primary	854 [15.61%]	134 [21.03%]	720 [14.88%]	
Secondary	2,484 [53.33%]	236 [42.84%]	2,248 [54.75%]	
Higher	407 [9.33%]	6 [2.02%]	401 [10.31%]	
Marital Status				
Not married	1,706 [39.35%]	270 [52.72%]	1,436 [37.54%]	< 0.0001
Married	3,596 [60.65%]	398 [47.28%]	3,198 [62.46%]	
Religion				
Christianity	3,314 [70.14%]	378 [67.27%]	2,936 [70.52%]	< 0.0001
Islam	1,707 [24.50%]	199 [18.51%]	1,508 [25.31%]	
Traditional/Other	281 [5.36%]	91 [14.23%]	190 [4.16%]	
Household Income				
Poor	2,342 [44.22%]	520 [63.19%]	2,481 [41.66%]	< 0.0001
Middle	1,054 [19.95%]	92 [21.68%]	871 [19.72%]	
Rich	1,906 [35.83%]	56 [15.13%]	1,282 [38.62%]	
Residence				
Urban	2,188 [48.00%]	189 [35.21%]	1,999 [49.73%]	0.0001
Rural	3,114 [52.00%]	479 [64.79%]	2,635 [50.27%]	
Health Insurance				
No health insurance	278 [4.95%]	95 [14.17%]	183 [3.71%]	< 0.0001
Has health insurance	5,024 [95.05%]	573 [85.83%]	4,451 [96.29%]	
Health Status				
Good	4,299 [79.67%]	517 [74.92%]	3,782 [80.31%]	0.0229
Moderate	819 [17.04%]	112 [19.54%]	707 [16.71%]	
Poor	184 [3.29%]	39 [5.54%]	145 [2.98%]	
Pregnancy				
No loss	4,063 [72.47%]	537 [73.54%]	3,526 [72.33%]	0.1076
1 loss	958 [20.55%]	108 [22.25%]	850 [20.32%]	
2 + losses	281 [6.98%]	23 [4.21%]	258 [7.35%]	

*All percents are column percents. Values are presented as weighted sample counts (N) and weighted column percentages (%)

*Percentages may not sum to 100 due to rounding

Across household income levels, the highest proportion of ANC users came from poor households (41.66%), followed by rich households (38.62%) and middle-income households (19.72%). Among different residence types, slightly more ANC users lived in rural areas (50.27%) than in urban areas (49.73%). Most women with health insurance (96.29%) used ANC, while only 3.71% without insurance did not. 

Regarding health status, the majority of women with good health (80.31%) had used ANC, followed by those with moderate health (16.71%) and those with poor health (2.98%). Similarly, most women who reported no pregnancy loss (72.33%) had used ANC, followed by those with a history of one loss (20.32%), and two or more losses (7.35%).

### Adjusted modified Poisson regression

Women aged 25–34 were more likely to use ANC compared to those aged 15–24 ( aPR = 1.04; 95% CI: 1.00-1.07; *p* = 0.035), whereas the association for women aged 35–49 was not statistically significant ( aPR = 1.03; 95% CI: 0.99–1.07; *p* = 0.129). Education showed an association: women with secondary ( aPR = 1.09; 95% CI: 1.05–1.14; *p* = < 0.0001) and higher education ( aPR = 1.10; 95% CI: 1.04–1.15; *p* = < 0.0001) were more likely to receive ANC compared to those with no education. Primary education was not associated with ANC utilization ( aPR = 1.04; 95% CI: 0.99–1.09; *p* = 0.14). Married women were also more likely to use ANC than unmarried women ( aPR = 1.07; 95% CI: 1.03–1.10; *p* = < 0.0001). Women who identified as Muslim were more likely to use ANC than Christian women ( aPR = 1.04; 95% CI: 1.01–1.07; *p* = 0.014), whereas those practicing traditional or other religions were less likely to use ANC services ( aPR = 0.83; 95% CI: 0.75–0.92; *p* = 0.001). Women from rich households have higher ANC use than those from poor families ( aPR = 1.08; 95% CI: 1.04–1.12; *p* = < 0.001). There was no difference between women from middle-class households and those from poor families. Type of residence (rural vs. urban) was not a predictor of ANC use. Compared to those who were uninsured, women with insurance were significantly more likely to use ANC services ( aPR = 1.29; 95% CI: 1.14–1.46; *p* = < 0.0001). Self-reported health status and pregnancy loss were not statistically significant with ANC utilization (See Table 3).

**Table 3:** Crude and adjusted prevalence ratios of antenatal care use by socio-demographic factors

Predictors	Antenatal Care Crude PR (95% CI)	*P*-Value	Antenatal Care Adjusted PR (95% CI)	*P*-Value
Age				
15 to 24	Ref.		Ref.	
25 to 34	1.08 [1.04, 1.12]	< 0.0001	1.04 [1.00, 1.07]	0.035
35 to 49	1.05 [1.01, 1.10]	0.008	1.03 [0.99, 1.07]	0.129
Education				
None	Ref.		Ref.	
Primary	1.03 [0.98, 1.09]	0.271	1.04 [0.99, 1.09]	0.14
Secondary	1.11 [1.06, 1.17]	< 0.0001	1.09 [1.05, 1.14]	< 0.0001
Higher	1.20 [1.13, 1.26]	< 0.0001	1.10 [1.04, 1.15]	< 0.0001
Marital Status				
Not married	Ref.		Ref.	
Married	1.08 [1.04, 1.12]	< 0.0001	1.07 [1.03, 1.10]	< 0.0001
Religion				
Christianity	Ref.		Ref.	
Islam	1.03 [1.00, 1.06]	0.06	1.04 [1.01, 1.07]	0.014
Traditional/Other	0.77 [0.68, 0.87]	< 0.0001	0.83 [0.75, 0.92]	0.001
Household Income				
Poor	Ref.		Ref.	
Middle	1.05 [1.00, 1.11]	0.073	1.02 [0.97, 1.08]	0.454
Rich	1.14 [1.10, 1.19]	< 0.0001	1.08 [1.04, 1.12]	< 0.0001
Residence				
Urban	Ref.		Ref.	
Rural	0.93 [0.90, 0.97]	< 0.0001	1.00 [0.97, 1.05]	0.831
Health Insurance				
No health insurance	Ref.		Ref.	
Has health insurance	1.35 [1.17, 1.57]	< 0.0001	1.29 [1.14, 1.46]	< 0.0001
Health Status				
Good	Ref.		Ref.	
Moderate	0.97 [0.93, 1.01]	0.183	0.98 [0.95, 1.02]	0.305
Poor	0.90 [0.81, 1.00]	0.059	0.96 [0.87, 1.05]	0.377
Pregnancy				
No loss	Ref.		Ref.	
1 loss	0.99 [0.95, 1.03]	0.652	0.98 [0.94, 1.02]	0.249
2 + losses	1.06 [1.02, 1.10]	0.006	1.02 [0.98, 1.06]	0.3

## Discussion

Using the Andersen Behavioural Health Model, our study examined how predisposing, enabling, and need factors are associated with ANC utilization among Ghanaian women. We found that age, education, marital status, religion, household income, and health insurance coverage were associated with ANC utilization. Women with secondary or higher education had a higher likelihood of ANC use, highlighting the role education plays in maternal health services utilization. Additionally, married women and those from wealthier households were more likely to use ANC services than their counterparts, indicating that social and economic support play a role in care-seeking. Women with health insurance also demonstrated a higher likelihood of ANC use, underscoring the importance of access to maternal health services. For age, only women aged 25–34 were slightly more likely to use ANC than those aged 15–24, with no significant difference among women aged 35–49. For religion, women who identified as Muslim were more likely to utilize ANC services compared to Christians. In contrast, women who identified with traditional or other religions are less likely to use ANC, suggesting potential religious preferences may be involved in decisions for maternal health-seeking care. Overall, our findings underscore the continued need for targeted interventions that address disparities across different demographic characteristics beyond overall ANC service availability.

Our findings offer new insights into how maternal age, a key predisposing factor, is associated with ANC utilization in Ghana. Prior studies have shown that age tends to have a limited influence on ANC use, with some reporting no differences or weak associations in service uptake [10, 19–21]. Our study echoes this pattern while adding nuance by showing variation within the reproductive age range. Specifically, women aged 25–34 showed a moderately strong, statistically significant association with ANC use, whereas women aged 35–49 did not. These results suggest that the influence of age on ANC utilization may be concentrated in early to mid-adulthood, whereas other socioeconomic or contextual factors may play a greater role for older women. One reason older women may not seek maternal health services as frequently as middle-aged women is their accumulated experience, which may foster a sense of self-reliance and reduce the perceived need for medical oversight. They may also receive different cues from healthcare providers, who might not emphasize ANC as strongly for this group. Furthermore, older women may face changes in social or household support systems that influence their care-seeking behaviour. These factors underscore the need to investigate in greater depth the unique circumstances that limit ANC use among older women.

Similar to existing literature, our studies found that women with higher education and are married are more likely to use ANC [21–23]. For example, Aboagye et al. found that women with higher education have two times higher odds of fulfilling the ANC requirements compared to those with no education [23]. Women with higher education may have greater knowledge of pregnancy, higher income, and greater autonomy in health care decision-making. Additionally, married women may benefit from greater social and financial support, which may facilitate maternal care utilization. These findings reinforce the importance of strengthening health education and empowering women to financial independence.

In contrast to earlier studies showing that Muslim women were less likely to seek maternal health services [24, 25], our findings indicate that Muslim women in Ghana were more likely to utilize ANC. One possible explanation is the Community-based Health Planning and Services (CHPS) program, which was established to improve rural access to preventive and ANC services [26]. The CHPS program originated in northern Ghana, an area with a high concentration of Muslim communities. This localized access may help explain the higher prevalence of ANC utilization among Muslim women in our study.

Among the enabling factors, household income and health insurance were associated with higher ANC use, aligning with previous research. Women with higher incomes may experience fewer financial barriers, such as transportation costs or indirect service fees. Similarly, having health insurance can reduce out-of-pocket expenses. Ultimately, these two enabling factors provide easier access to greater ANC utilization. However, despite residence being a key enabling factor, our analysis did not observe differences between urban and rural women in ANC utilization, a finding that contrasts with existing studies. This can also be explained by the long-term expansion of the CHPS program, which has been implemented for more than two decades and has significantly increased access to maternal health services in rural communities [26]. This sustained expansion may have improved the traditional urban–rural disparities in ANC use. Overall, our findings regarding the higher ANC utilization among Muslim women and the lack of differences between urban and rural women suggest that national programs may be contributing to more equitable access to maternal health services in Ghana.

Beyond sociodemographic characteristics, we also examined need factors that determine whether individual health factors play a role in ANC utilization. Our analysis did not show an association for women’s self-reported health status or history of pregnancy loss with ANC utilization. One possible explanation is that ANC attendance in Ghana may be shaped by socioeconomic factors, such as access to care, education, and wealth, rather than by an individual’s health perception. The lack of association is important because it suggests that ANC uptake is not driven by perceived need, underscoring the need to continue addressing sociodemographic disparities for attaining optimal maternal health outcomes.

### Implications

Our findings highlight two key policy implications for strengthening maternal health care in Ghana. First, given that women with higher education use ANC more, it is crucial to continue investing in girls’ education and adult literacy programs. Second, the strong association between health insurance coverage and ANC use emphasizes the importance of sustaining and expanding the NHIS program. This would help to ensure that financial barriers do not prevent pregnant women from seeking maternal health care. Furthermore, the absence of urban–rural differences in ANC utilization suggests that long-term national initiatives, such as CHPS, are effective in reducing geographic disparities.

### Limitations

While our study offered new insights into factors associated with ANC utilization, a few limitations should be acknowledged. First, because longitudinal data are unavailable, the data are cross-sectional and cannot demonstrate causal effects. Additionally, the Andersen model applied here does not account for socio-cultural influences, such as traditional norms, gendered decision-making, and perceptions of pregnancy risk, that often act as barriers to ANC utilization. Future research could address these gaps by using longitudinal data and integrating qualitative or mixed-methods designs. Additionally, factors such as spousal approval, social stigma, cultural norms, and support networks are critical to understanding maternal health-seeking behaviours. Such approaches would allow for a deeper exploration of persistent disparities in ANC use, even in the context of Ghana’s free, universal maternal health policies.

## Conclusion

Our study showed that age, education, marital status, religion, household income, and health insurance coverage were associated with ANC utilization. These findings highlight the need to strengthen education, financial access, and insurance coverage to ensure equitable utilization of maternal healthcare. Additionally, culturally informed approaches are essential to improving maternal healthcare access. Future research should conduct qualitative research to capture socio-cultural barriers that remain invisible within DHS data. In particular, future studies should investigate why women still forgo ANC despite the 2008 policy of free maternal health services.

## Supplementary Information

Supplementary Material 1.

## Data Availability

The data supporting this study are available from the corresponding authors upon reasonable request.

## Abbreviations

APR: Adjusted Prevalence Ratio
ANC: Antenatal Care
CI: Confidence Interval
DHS: Demographic and Health Survey
GDHS: Ghana Demographic and Health Survey
VIF: Variance Inflation Factor
PR: Prevalence Ratio
NHIS: National Health Insurance Scheme
CHPS: Community-based Health Planning and Services
