# Supplementary material for: Social determinants of antenatal care utilization: an analysis of 2022 Ghana demographic and health survey
Source: BMC Pregnancy Childbirth. 2026 Jan 12;26:226. doi: 10.1186/s12884-025-08632-7 (PMC12958708; doi:10.1186/s12884-025-08632-7)
Supplement: Supplementary file 1 — Supplementary Material 1. [file 12884_2025_8632_MOESM1_ESM.docx]

| **Supplementary Table 1.** Variance Inflation Factors (VIF) | | |
| --- | --- | --- |
| Variable | **VIF** | **1/VIF** |
| Age |  |  |
| 15 to 24 | Ref. | Ref. |
| 25 to 34 | 1.67 | 0.598 |
| 35 to 49 | 1.77 | 0.564 |
| Education |  |  |
| None | Ref. | Ref. |
| Primary | 1.44 | 0.697 |
| Secondary | 1.96 | 0.509 |
| Higher | 1.60 | 0.624 |
| Marital Status |  |  |
| Not married | Ref. | Ref. |
| Married | 1.29 | 0.776 |
| Religion |  |  |
| Christianity | Ref. | Ref. |
| Islam | 1.27 | 0.787 |
| Traditional/Other | 1.12 | 0.896 |
| Household Income |  |  |
| Poor | Ref. | Ref. |
| Middle | 1.35 | 0.739 |
| Rich | 1.92 | 0.522 |
| Residence |  |  |
| Urban | Ref. | Ref. |
| Rural | 1.5 | 0.667 |
| Health Insurance |  |  |
| No health insurance | Ref. | Ref. |
| Has health insurance | 1.03 | 0.973 |
| Health Status |  |  |
| Good | Ref. | Ref. |
| Moderate | 1.02 | 0.977 |
| Poor | 1.03 | 0.975 |
| Pregnancy |  |  |
| No loss | Ref. | Ref. |
| 1 loss | 1.05 | 0.954 |
| 2+ losses | 1.05 | 0.949 |
|  |  |  |
| Mean VIF = 1.38 |  |  |
